# RED BLOOD CELL DISTRIBUTION WIDTH: FURTHER INSIGHTS

**DOI:** 10.1093/geroni/igae098.4062

**Published:** 2024-12-31

**Authors:** Matt Oberdier, Marta Zampino, Michelle Shardell, Majd AlGhatrif, Edward Lakatta, Eleanor Simonsick, Luigi Ferrucci

**Affiliations:** National Institute on Aging, Baitmore, Maryland, United States; University of Maryland, Baltimore, Maryland, United States; University of Maryland School of Medicine, Baltimore, Maryland, United States; Johns Hopkins University, Baltimore, Maryland, United States; National Institute on Aging, Baitmore, Maryland, United States; National Institute on Aging, Baitmore, Maryland, United States; National Institute on Aging, Baitmore, Maryland, United States

## Abstract

Red blood cell distribution width (RDW) measures erythrocyte size variability and strongly predicts cardiovascular disease (CVD) morbidity and mortality. Our prior cross-sectional analysis showed that inflammation, metabolic rate, and body habitus are independently associated with RDW. To better understand determinants of RDW, we performed a longitudinal analysis associating changes in RDW within the normal range (11.5 - 15.0%) with age, sex, race, and changes in other erythrocyte properties, major CVD risk factors, and other physiologic measures. Data were from 452 men and 482 women, aged 50 to 94 years enrolled in the Baltimore Longitudinal Study of Aging. For each participant and each predictor, change was the regression coefficient of time from a linear model. Coefficients of candidate correlates were then used to predict change in RDW in multiple linear regression models that were reduced by backward selection. In the final model, increasing erythrocyte number (STβ=1.4980, P< 0.001), mean platelet volume (STβ=0.2019, P< 0.001), anion gap (STβ=0.1754, P< 0.001), blood urea nitrogen (STβ=0.0684, P=0.0395), urine albumin-creatinine ratio (STβ=0.1007, P=0.0015), alanine transaminase (STβ=0.1580, P< 0.001), interleukin-6 (STβ=0.2338, P< 0.001), and diastolic blood pressure (STβ=0.0769, P=0.0107) and decreasing glucose (STβ=-0.2425, P< 0.001), vitamin B12 (STβ=-0.1310, P< 0.001), and C-reactive protein (STβ=-0.1801, P< 0.001) were associated with increasing RDW. The association of age with increasing RDW was independently explained by decreasing glucose (STβ=-0.2310, P< 0.001). Taken together, these findings suggest that rising RDW may reflect accelerated aging. Studies in larger, more diverse populations are necessary to confirm these findings and determine their role in mechanisms leading to age-associated increases in RDW.

